# A Genetic Association Study of *MTHFR* C677T Polymorphism with Risk of Metabolic Syndrome: A Systematic Review and Meta-Analysis

**DOI:** 10.31661/gmj.v8i0.1472

**Published:** 2019-06-02

**Authors:** Soheil Azizi, Amir Shamshirian, Reza Alizadeh-Navaei, Hamed Jafarpour, Zatollah Asemi, Omid Reza Tamtaji, Mohammad Sadegh Vaziri, Reza Homayounfar, Arash Rezaei Shahmirzadi, Reza Alipoor

**Affiliations:** ^1^Department of Laboratory Sciences, Faculty of Paramedicine, Mazandaran University of Medical Sciences, Sari, Iran; ^2^Student Research Committee, Mazandaran University of Medical Sciences, Sari, Iran; ^3^Gastrointestinal Cancer Research Center, Mazandaran University of Medical Sciences, Sari, Iran; ^4^Research Center for Biochemistry and Nutrition in Metabolic Diseases, Kashan University of Medical Sciences, Kashan, Iran; ^5^Physiology Research Center, Kashan University of Medical Sciences, Kashan, Iran; ^6^Student Research Committee, Hormozgan University of Medical Sciences, Bandar Abbas, Iran; ^7^Non-Communicable Disease Research Center, Fasa University of Medical Sciences, Fasa, Iran; ^8^Student Research Committee, Golestan University of Medical Sciences, Gorgan, Iran

**Keywords:** *MTHFR*, Metabolic Syndrome, Polymorphism, Variant, Meta-Analysis, Methylenetetrahydrofolate Reductase

## Abstract

Methylenetetrahydrofolate reductase (*MTHFR*) is an enzyme that plays a crucial role as a methyl-group donor in demethylation of homocysteine. The aim of this systematic review and meta-analysis was to study the relationship between *MTHFR* gene polymorphism and metabolic syndrome (MS). We used search engines and databases such as Science Direct, Google Scholar, Embase, Cochrane Library, and PubMed to identify eligible studies up to 2018. The articles were studied based on keywords including *MTHFR*, mutation, variant, and polymorphism in combination with MS. Data was analyzed using Comprehensive Meta-Analysis version 2.2.064 software. After extracting the data from seven articles, the total number of subjects was 1280 in the patient group and 1374 in the control group. The odds ratio was estimated to be 1.078 for the allele model of T vs. C (95% confidence interval [CI]: 1.626-0.715), 1.157 for the allele model of CC vs. CT (95% CI: 0.829-1.615), 1.020 for the allele model of CT + TT vs. CC (95% CI: 1.611-0.646) and 0.799 for the allele model of TT vs. CC + CT (95% CI: 1.185- 0.539). As well, the results showed no statistically significant correlation between polymorphism genotypes of the *MTHFR* gene and MS (P<0.05). In general, this study showed that the presence of C677T polymorphism in the *MTHFR* gene has no effect on the incidence of MS.

## Introduction

Methylenetetrahydrofolate reductase (*MTHFR*) is an enzyme that plays an important role as a methyl-group donor in demethylation of homocysteine [1]. Due to the incidence of C677T mutation in the *MTHFR* gene, thymine is replaced by cytosine, followed by the translation of valine instead of alanine in the structure of the produced enzyme, resulting in the formation of a temperature-sensitive enzyme and thus reducing its activity [2]. The set of these changes increases the concentration of homocysteine and endothelial dysfunction and accelerates the oxidation of lipoproteins [3]. It has been observed that the levels of homocysteine increase in patients with diabetes mellitus (DM) [4, 5] and hypertension [6, 7]. Some studies also pointed to the relationship between hyperhomocysteinemia and insulin resistance [8-10]. This relationship can be partially justified in light of the correlation of C677T polymorphism (occurring in the *MTHFR* gene) with hypertension [11], DM [12, 13], and diabetic nephropathy [14]. In recent years, there has been a significant relationship between *MTHFR* and metabolic syndrome (MS) in certain groups of people with type 2 DM [15] and schizophrenia [16], but these studies have very different results in relation between *MTHFR* and MS, and no comprehensive study has been done to summarize these outcomes in patients with MS. Regarding the high prevalence of MS and the role of genetic factors in the disease [17], determining the relationship between *MTHFR* polymorphism and MS can be helpful. In this way, people who are genetically predisposed to the disease can be identified more quickly to fulfill preventive interventions. The aim of this systematic review and meta-analysis was to study the relationship between *MTHFR* gene polymorphism and MS.

## Search Strategies

Search engines and databases including PubMed, Embase, ScienceDirect, Cochrane Library, and Google Scholar were searched to find all English articles up to 2018. The related keywords were extracted using the Medical Subject Heading (MeSH), containing “*MTHFR*,” “mutation,” “variant,” and “polymorphism” in combination with “MS.” Also, the search was also conducted in the language restriction. Two of the authors reviewed the articles considering the inclusion and exclusion criteria, and the third author reviewed controversies to take the final decision. All articles aiming to investigate the relationship between *MTHFR* polymorphisms and MS were introduced into the study. Selected articles for meta-analysis had the following features: evaluation of the relationship between *MTHFR* gene and MS, number of subjects in case and control groups, access to distribution of genotypes and alleles in case and control groups, considering 95% confidence interval (CI) to estimate odds ratio (OR), original research articles, randomized and controlled articles, and considering gene polymorphism as the main independent variable. Also, when several investigations were conducted on the same population, the latest study was selected to enter into the meta-analysis. The standard information form was used for data collection. Form information included the author name, publication year, study location, genotype type, total number of subjects in case and control groups, genotype distribution in the case and control groups, and the frequency of the dominant allele in both case and control groups. Two of the authors performed the process of extracting information from articles. Hardy-Weinberg equilibrium, genotyping methods qualification, controls source, sample size, and cases representativeness, were examined with a total score of 10 in this scoring. This checklist had been used in previous studies. Besides, scores zero to four were categorized as a weak study, five to seven as an average study, and eight to 10 as a strong article. The allele frequency for gene polymorphism was determined in each study using the allele counting method. OR with 95% CI was used to evaluate the power of correlation between *MTHFR* gene and MS, followed by the allele model (C vs. T), multiplicative model (CC vs. TT), the dominant model (CC + CT vs. TT), and recessive model (CC vs. CT + TT). Heterogeneity was calculated using measurement test and the random effects model. The I-square (*I2*) index of 25, 50, and 75 percent showed lower, moderate, and high levels of heterogeneity, respectively. The bias of published articles was examined using a funnel plot versus standard error (SE). Publication bias was evaluated using both Begg’s funnel plot and Egger’s linear regression test. Because of significant heterogeneity of the results (*I2*>30%), the random effects model, which takes the diversity of the studies into account, was used. All analyses were performed by Comprehensive Meta-Analysis version 2.2.064 (CMA) software. *A*P-value of less than 0.05 was considered as a significance level.

## Results

**Figure 1:**
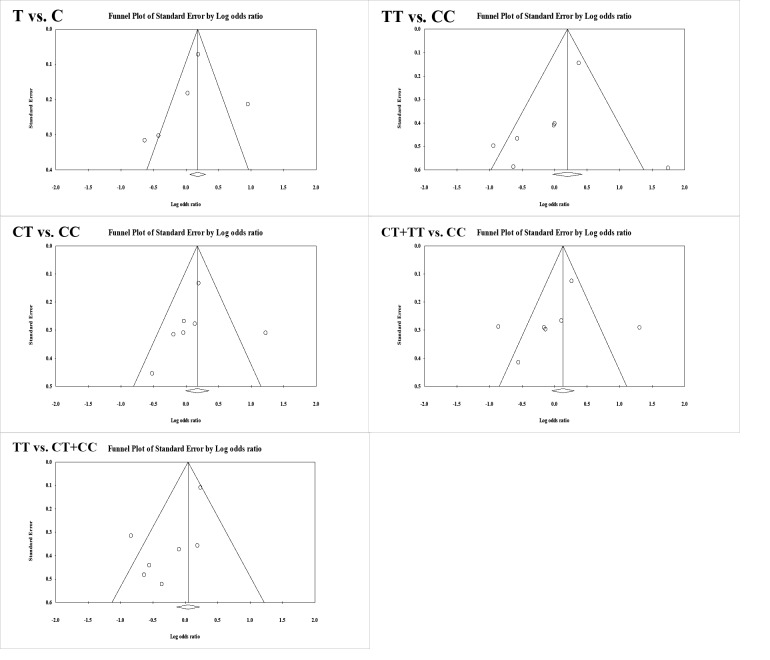
Funnel plot of standard error by log odds ratio

**Figure 2:**
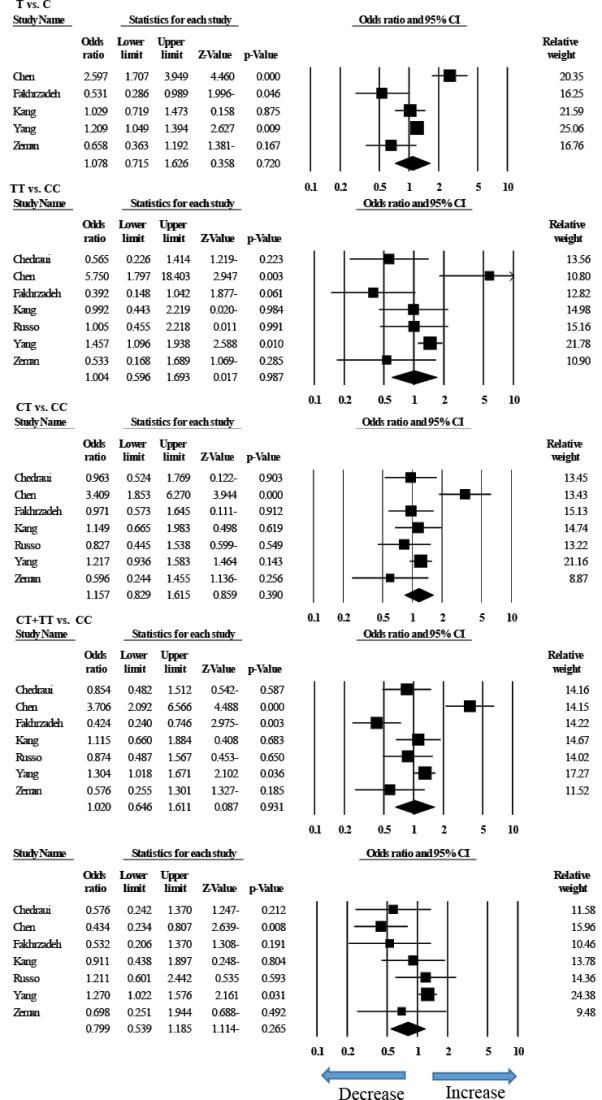
Forest plot analysis for assessing the overall MS risk associated with C677T polymorphism

In total, 95 studies were systematically reviewed. Some studies were excluded because of the similarity of the studied samples (n=69), the inappropriateness of the samples and the failure to consider the MS as the main variable (n=22), and inadequate information on alleles (n=4). Finally, seven papers [18-23] were entered into the meta-analysis. Studies characteristics are presented in Table-1. To evaluate the publication bias of studies entered into the meta-analysis, Egger’s test and Begg’s funnel plot were used (Table-2). In all genetic models, the appearance of the shape of the funnel plots was symmetrical (Figure-1). We used Egger’s test to provide the statistical evidence of funnel plot. The findings of the research showed that there is no publication bias in comparison models. Heterogeneities were evaluated by using an *I2* test and *Q*-test. Heterogeneity was observed in all the models, i.e., allele (T vs. C), homozygous (TT vs. CC), heterozygous (CT vs. CC), dominant (CT + TT vs. CC), and recessive (TT vs. CT + CC) genotype model, which were included for the meta-analysis. Therefore, the random effects model, which takes the diversity of the studies into account was used for data analysis. Meta-analysis of C677T polymorphism in the *MTHFR* gene and MS in total consisted of six case-control studies and one cohort study. Available studies in the meta-analysis were used to evaluation of the relationship between *MTHFR* polymorphism and MS. In general, these studies showed that the total number of subjects was 1280 in the MS patient group and 1374 in the healthy control group. According to the findings of this study (Figure-2), there was no significant relationship between *MTHFR* 677 C> T polymorphism and MS in the allele model (T vs. C: P= 0.720; OR = 1.078, 95% CI = 0.715 to 1.626), in the homozygous model (TT vs. CC: P= 0.987, OR = 1.004, 95% CI = 0.596 to 1.693), in the heterozygous model (CT vs. CC: P= 0.390, OR = 1.157, 95% CI = 0.829 to 1.615 ), in the dominant model (CT + TT vs. CC: P= 0.931, OR = 1.020, 95% CI = 0.646 to 1.611) and in the recessive model (TT vs. CT + CC: P= 0.265; OR = 0.799, 95% CI = 0.539 to 1.185).

## Discussion

According to the findings of this study, there was no significant relationship between C677T polymorphism in the *MTHFR* gene and MS. According to the C677T polymorphism in the *MTHFR* gene and MS, the result of the present study is in the same line with a study carried out by Russo *et al*. [15]. They indicate that there is no association between the *MTHFR* polymorphism and MS in patients with type 2 DM with mild hyperhomocysteinemia [15]. Yamada *et al*. [24] examined the possible gene responsible for the incidence of MS in 1,788 Japanese individuals, and they found no link between the *MTHFR* polymorphism and the prevalence of MS. However, Ellingrod *et al*. observed that the CT mutation in the *MTHFR* gene predisposes those with schizophrenia taking atypical antipsychotics to MS [25]. This inconsistency is partially justified by epigenetic mechanisms. In this regard, there is a hypothesis that, in addition to inheriting the thrifty gene, epigenetic mechanisms also affect embryonic and postnatal development, and MS underlying disease including insulin resistance, local obesity, dyslipidemia, and hypertension [26]. This generation may even inherit these mechanisms from their fathers or grandparents. Since mitosis occurs during adulthood, epigenetic pathways can affect the expression of the gene in all stages of life. The *MTHFR* enzyme acts as a methyl-group donor for the remethylation of homocysteine and its conversion into methionine. Methionine consumes methyl group for DNA methylation, especially in CpG pairs. These pairs, which exist in certain regions, act as a promoter for related genes [27]. As a result, environmental and nutritional factors can affect the relationship between *MTHFR* and MS through these epigenetic mechanisms [28]. It is necessary to carry out comprehensive demographic studies to confirm the conclusion of the present study. According to the results, it is recommended that the necessary interventions should be promoted to change lifestyles to modify the epigenetic mechanisms in society. Flour fortification with folic acid is one of the best available actions. Hypotheses suggest that folic acid fortification can overcome the metabolic block resulting from *MTHFR* mutation and subsequently affect DNA methylation and gene expression. We had some limitations in this study. These limitations included lack of access to some of the main articles in English and non-English languages.

## Conclusion

In general, this study showed that the presence of C677T polymorphism in the *MTHFR* gene has no effect on the incidence of MS. It is suggested to evaluate the effect of folic acid fortification and supplementation on the expression of the *MTHFR* gene, in particular, those associated with chronic diseases such as hypertension, DM, and MS.

## Acknowledgment

We are sincerely thankful to our counsellors in Clinical Research Development Center of Shahid Mohammadi Hospital. The study was approved by the ethics committee of Hormozgan University of Medical Sciences (grant number: 970386 and ethics committee registration code: IR.HUMS.REC.1397.327). We would like to thank all the people who helped us with collecting the data and the searching works.

## Conflict of Interest

None.

**Table 1:** Studies Characteristics and Distribution of C677T Polymorphism

**Variables** **Chedraui** ***et al***.		**Authors**						
		**Chen** ***et al***.	**Fakhrzadeh** ***et al***.	**Kang** ***et al***.	**Russo** ***et al***.	**Yang** ***et al***.	**Zeman** ***et al***.	
**Reference**		[18]	[19]	[20]	[21]	[15]	[22]	[23]
**Year**		2012	2008	2009	2009	2002	2011	2008
**County of region**		Ecuador	China	Iran	Korea	Italy	China	Prague
**Study design**		PB	PB	PB	HP	HB	PB	PB
**Genotyping method**		PCR	PCR	PCR	PCR	PCR	PCR	PCR
**Cases**		103	118	150	110	50	692	57
**Controls**		89	95	76	145	50	878	41
**Cases**	**CC**	48	34	102	36	36	129	30
	**CT**	45	61	38	60	49	335	19
	**TT**	10	23	10	14	21	228	8
**Control**	**CC**	38	57	36	51	31	202	16
	**CT**	37	30	31	74	51	431	17
	**TT**	14	8	9	20	18	245	18
**Minor allele frequency (Cases)**		0.315	0.466	0.193	0.4	0.429	0.571	0.307
**Minor allele frequency (Controls)**		0.365	0.242	0.322	0.393	0.435	0.524	0.519
**HWE (P-value)**		0.329	0.173	0.562	0.402	0.707	0.638	0.017

**Table 2:** Statistics to Test Publication Bias and Heterogeneity in the Meta-Analysis

**Comparison Model**	**Egger’s regression analysis**			**Heterogeneity analysis**			**Model used for the meta-analysis**
	Intercept	95% confidence interval	P-value	*Q*-value	*P*_(Heterogeneity)_	*I* ^2^(%)	
**T vs. C**	-1.05	-8.21 to 6.10	0.670	24.27	<0.001	83.521	Random
**TT vs. CC**	-1.26	-4.52 to 1.99	0.362	18.66	0.005	67.852	Random
**CT Vs. CC**	-0.60	-5.14 to 3.93	0.745	16.16	0.013	62.880	Random
**CT+TT vs. CC**	-1.79	-7.98 to 4.39	0.489	33.68	<0.001	82.190	Random
**TT vs. CT+CC**	-2.00	-3.97 to 2.62	0.046	15.49	0.017	61.286	Random
